# In Pursuit of the Most Effective Method of Teaching Feedback Skills to Emergency Medicine Residents in Qatar: A Mixed Design

**DOI:** 10.7759/cureus.8155

**Published:** 2020-05-16

**Authors:** Khalid Bashir, Amr Elmoheen, Mohammed Seif, Shahzad Anjum, Saleem Farook, Stephen Thomas

**Affiliations:** 1 Emergency Medicine, Hamad Medical Corporation, Doha, QAT; 2 Medicine, Qatar University, Doha, QAT

**Keywords:** feedback skills, medical education, emergency medicine

## Abstract

Purpose

The study aimed to find an effective method of teaching feedback skills to residents and to gauge their preference.

Method

This was a mixed design study conducted at the emergency department of a large tertiary care hospital. The residents were randomized to groups A, B, and C. Group A (control) received a traditional lecture, Group B read a specifically written brief document, and Group C received 1:1 tutoring from one faculty. Each resident individually watched a four-minute video on an emergency procedure and provided feedback in simulated settings, which was audio-recorded and rated by two blinded raters. An assessment form was created and validated. The residents’ preference was attained through a semi-structured interview.

Results

The baseline characteristics of the three groups were similar. Compared to Group A, Groups B and C scored significantly higher on the overall assessment and were statistically similar to each other. There was no sign of association between both gender and postgraduate score (PGY) year on the total score. Residents’ equally preferred self-reading and 1:1 tutoring.

Conclusion

The acquisition of feedback skills by emergency medicine (EM) residents was comparable between self-learning from an appropriately written document and 1:1 teaching by adequately trained faculty.

## Introduction

Constructive feedback provided by appropriately trained faculty is a potent instrument in medical education. It helps the learners to comprehend strengths, understand limitations, and provide a roadmap for future progress in their clinical career. Poor feedback may result in errors not being corrected and good performance not being recognized as potentially impacting clinical performance [1]. Veloski and colleagues conducted a systematic review and found a positive correlation between feedback and clinical performance [2].

The residency curriculum requires future residents to be effective teachers who have competent feedback skills [3]. The residents are more likely to respond to feedback by experienced and appropriately trained faculty as compared to other instructions [4]. A study among medical students confirmed that there was a lack of effective feedback in almost half of the interactions due to the poor feedback skills of the clinical faculty and the absence of a clear feedback system [5]. In spite of the widespread recommendation of constructive feedback in the education literature, the feedback provided is of low quality [6]. One of the concerning aspects is that feedback in medical education is provided by faculty with little or no formal training in this teaching role [6]. Poor or inappropriate feedback is not only present in clinical medicine but is also rife in business administration, psychology, and educational literature [7]. Specific training may be required to provide effective feedback. In one study, a postgraduate qualification in medical education did not endorse the faculty’s ability to provide appropriate and constructive feedback [8].

There are several reasons why effective feedback does not happen. Some tutors feel that negative feedback may not help students to progress and damage their educational relationships [9]. Some teachers are reluctant to convey corrective feedback, particularly in front of patients and colleagues. At the same time, trainees report reluctance in asking for feedback, which if critical might make them defensive and may make the matter worse [10]. Lack of knowledge and training of the clinical faculty has also been cited as an important barrier to effective feedback [11].

While the literature supports the training of medical educators on how best to give effective feedback, there is no rigorous study recommending the best methods to improve the quality of such feedback [12-13]. Previous studies lack randomization and consist of small, heterogeneous study populations. In order to enhance the quality of feedback, such training should be undertaken during residency [14]. In the host institution, the teaching of feedback skills to the emergency medicine residents (EMR) is provided by emergency physicians (EP) through traditional lectures (TL) and small group teachings during normal didactic teaching days. TL has been criticized in the past due to difficulty in maintaining attention for more than 10-15 minutes [15]. The study aimed to compare the effectiveness of self-learning and 1:1 tutoring as compared to the standard lecturing in acquiring feedback skills among emergency medicine (EM) residents in Qatar. The secondary aim was to gauge their preference for an educational method.

## Materials and methods

Institutional ethics approval was obtained from the Medical Research Center (MRC) of Hamad Medical Corporation (MRC-01-18-136), and the study was classified as exempt category 1, research conducted in the established commonly accepted educational settings involving normal educational practices. MRC also funded the project.

Setting

The study was conducted in the largest teaching hospital in Doha, the capital of the state of Qatar. The host hospital emergency department sees approximately half a million patients every year. The study center runs a four-year emergency medicine residency (EMR) training program, which has been accredited by the international branch of the USA-based Accreditation Council for Graduate Medical Education. All residents attend their didactic educational training on Tuesday every week, and the study was conducted during these days in the months of March and April 2019.

Software package

The information from the study was recorded into a dataset in the statistical package Stata (version 15MP, Stata Corp, College Station, Texas). Stata was used for all graphing and analytics for this report.

Design

This was a mixed design study. The research assistant, statistician, and the faculty responsible for rating were blinded to the type of educational intervention. Two experienced faculty members prepared a standardized clinical skill teaching video of approximately four minutes duration. The video demonstrated an often-performed clinical skill relevant to EPs. The procedure is endotracheal intubation on a manikin performed by a clinical fellow in EM and supervised by EM faculty. These roles are played by two senior faculty members involved in the study. The video was reviewed by a senior faculty member with experience in medical education and in giving feedback. Following this review, it was concluded that the video is a suitable demonstration of a skill on which feedback could be provided during the study.

Randomization and blinding

Each resident was randomized into one of three groups (A, B, or C) through the utilization of computer-generated block randomizations. The educational material presented to the residents was similar in all three groups. The randomization was prepared using computer-generated random numbers. The block sizes were 3, 6, and 9. The EM residents were assigned with equal probability to the three groups. The randomization sequence was concealed in envelopes that were sealed and opaque. Like most other educational trials, it was difficult to blind the residents and faculty; however, the outcome raters and statisticians were kept blinded to the study groups.

Control and intervention groups

Forty-five EMRs partook in the study and were in different years of training. Fifteen EMRs were assigned to group A (TL), group B (self-reading), and group C (1-1 teaching). Group A (as well as the two other groups) received one-hour teaching during their residency training within the last 12 months. This teaching consisted of a lecture followed by discussion in small groups. The intervention group (B) read one A4-page instruction, specially prepared based on the attributes of good feedback (Appendix A). The document was reviewed by two experienced physicians, who found it to be appropriate for residents’ training. Finally, the document was successfully piloted on two recently board-certified EMRs. During the trial, most residents read the document twice, and some made the notes; however, all the written notes were removed while watching the video and audio-recording their feedback. The intervention group (C) received one 1:1 teaching on the characteristics of good feedback by one experienced faculty. Each resident in group B and C spent 15-20 minutes on the study.

Validation of the study questionnaire

Based on recent literature, 10 characteristics were identified that should be present in a good feedback discussion [16]. Based on these characteristics, a 10-item questionnaire with a yes/no answer was prepared. A four-minute educational video was prepared in which one faculty member is providing feedback to the resident about his lecture on shock. The video aimed to facilitate the validation of the questionnaire. The video was shown as a group to 19 senior fellows and consultants, and they were asked to rate the 10-part questionnaire individually. If all the raters agreed that the person addressed the issue in the video then the overall agreement would be close to 100% percent, if everyone agreed that the person did not address the issue on the video, the overall proportion would be close to 0%. If the percentage was close to 100% or close to 0%, there was a good agreement. In eight out of 10 questions, there was virtually 100% agreement, one-sided 97.5% CI, for the agreement point estimate of 100% (19/19) was 82.4%-100%. The 10-item number questionnaire was used in the study (Appendix B).

Outcome measures

Each feedback was audio-recorded and was given a code and anonymized. Using digital media, such as video and audio recording, in medical education research has been recommended in previous studies [17]. Two blinded raters who are experienced faculty were trained and assessed for rating the audio recordings. In 43/45 recordings, there was 100% agreement between the two raters. In the remaining two audio recordings, there was only a 50% agreement; hence, these were rated by a third blinded faculty. During the semi-structured interview, eight EMRs were asked about their preference for one educational intervention.

Analysis of data

Categorical variables were reported as counts and proportions, while continuous variables were summarized as means with their corresponding standard deviations (SD). Fischer’s exact test was used to compare categorical variables, while the mean scores across various categories were compared using the one-way analysis of variance (ANOVA). The degree of association between significant variables on one-way ANOVA was further explored using simple linear regression analysis. The information was recorded into a dataset in the statistical package Stata (version 15MP, Stata Corp, College Station, Texas USA). Stata was used for all graphing and analytics for this report.

Ethical approval

The study was approved by the Medical Research Center (MRC) of the host hospital (RP-01-18-136).

Participants consent

The study was classified as exempt under the Supreme Council of Health Qatar guidelines.

Author’s contribution

KB and AE conceived the study, obtained ethical approval and wrote the first manuscript, MS and SA conducted the study, collected the data, and critically appraised the literature, SH and ST did the detailed statistical analysis, wrote the report, and performed overall supervision.

Conflict of interest

There was no conflict of interest.

## Results

A total of 45 EMR participated in the study; there was a difference in the number of males and females (Male = 68.9% vs Female = 31.1%; p=0.174) and postgraduate year (PGY) (PGY 1 = 28.9%, PGY 2 = 24.4%, PGY 3 = 26.7%, PGY 4 = 20.0%; p=0.530) across the different trial groups at baseline; however, it was not significantly different from the ratio of male and female EMRs in the institute where the study was conducted. The difference was also not relevant to the objectives and the results of the study with no significant difference in gender (Tables 1-2).

**Table 1:** Assessment of whether groups A, B, and C were similar in composition (by gender)

Gender	Group			Total
	A	B	C	
Female	2 (13.33%)	5 (33.33%)	7 (46.67%)	14 (31.11%)
Male	13 (86.67%)	10 (66.67%)	8 (53.33%)	31 (68.89%)
Total	15 (100.00%)	15 (100.00%)	15 (100.00%)	45 (100.00%)
Fisher’s exact = 0.174; No difference in gender across groups				

**Table 2:** Assessment of whether groups A, B and C were similar in composition (by PGY) PGY: postgraduate year

PGY	Group			Total
	A	B	C	
1	4 (26.67%)	4 (26.67%)	5 (33.33 %)	13 (28.89%)
2	5 (33.33 %)	2 (13.33%)	4 (26.67%)	11 (24.44%)
3	2 (13.33%)	7 (46.67%)	3 (20.00%)	12 (26.67%)
4	4 (26.67%)	2 (13.33%)	3 (20.00%)	9 (20.00%)
Total	15 (100.00%)	15 (100.00%)	15 (100.00%)	45 (100.00%)
Fisher’s exact = 0.530 No difference in PGY spread across groups				

There was a statistically significant difference in mean scores across the intervention groups on one-way analysis of variance (ANOVA) (Group A mean score [SD] = 3.5 [0.6]; Group B mean score [SD] = 4.8 [1.2]; and Group C mean score [SD] = 6.3 [1.2]; p=0.001) (Table 3).

**Table 3:** Total score across the three groups A, B, and C The one-way ANOVA assessment of the above indicated there was a statistically significant association between the group and the total score (p = 0.001). ANOVA: analysis of variance

	Number	Mean	Standard Deviation (SD)
Group A	15	3.533333	0.6399405
Group B	15	4.8	1.897367
Group C	15	6.266667	1.222799

However, no statistically significant difference in mean scores was noted across the PGY (p=0.594).

On simple linear regression, participants in groups B (b = 1.7, 95% confidence interval [CI] = 0.3 - 2.3, p= 0.014) and C (b = 2.7, 95% CI = 1.7 - 3.7, p= <0.001) had significantly higher mean scores as compared to those in Group A (no intervention). The regression model explained up to 42.1% of the total variability in mean scores across groups. The EMRs equally favored self-reading and 1:1 tutoring.

## Discussion

The aim of the RCT was to compare commonly employed educational methods of teaching feedback skills to EM residents. The study demonstrated that there was a statistically significant better performance in the overall assessment of both educational interventions’ groups (B & C) as compared to the control group (A). There was no sign of association between either sex or PGY year on total score.

There was a statistically significant improvement in scores in both intervention groups. On simple linear regression, participants in groups B (b = 1.7, 95% confidence interval [CI] = 0.3 - 2.3, p= 0.014) and C (b = 2.7, 95% CI = 1.7 - 3.7, p= 0.001) had significantly higher mean scores as compared to those in Group A (no intervention). Self-reading is a common method employed for acquiring knowledge and skills among the residents. Self-reading and making notes of the important and relevant points helps to retain the information. This technique has been recommended for medical students learning [18]. 1:1 teaching encourages active learning, which is an outstanding method of acquiring knowledge and skills but involves a substantial amount of faculty time [19]. During 1:1 teaching, the faculty can easily focus on the needs of the learner, and this close interaction raises the potential for an effective outcome [20]. Both these methods were preferred by residents, and a recommendation has been forwarded to the residency program for future consideration [16].

We have identified several limitations in our study. First, the study was conducted in a single academic center and the sample may not be representative of other sites; hence, the results may not be generalizable. The EMRs completed their four years of training within the host hospital. For this practical reason, only local recruitment was done. Second, only two educational methods were considered in this trial and compared with the traditional didactic lecture. There are other popular educational methods, such as blended learning, which may have provided a better outcome. Due to the limited time available during the residency educational days, these two methods were most appropriate for the study. Third, we only assessed the short-term effect of educational interventions. The long-term effects, possibly six or 12 months after the educational intervention, would have been more useful to look at retention of feedback skills. EM residents spend 18 months outside the department rotating to other specialties; hence, their availability for a follow-up study is not guaranteed. The fourth limitation was the limited use of semi-structured interviews for assessing the preference for educational methods. The use of the Focus group would have provided more detailed information. Due to the limited time available during the residents’ education days, we were not able to conduct detailed interviews.

## Conclusions

The study showed that either a carefully prepared article or 1:1 teaching resulted in an improvement of feedback-giving skills to EM residents in the short term. The study may have implications on the utilization of faculty time for teaching core skills to EMR. Future research should investigate the long-term retention of educational interventions.

## Appendices

Appendix A

Self-reading instructions

Please read the following instructions before watching the video and recording your feedback.

The following steps have been recommended in the literature for effective feedback:

1. Establish a respectful learning environment

2. Communicate goals and objectives for feedback

3. Make feedback timely and a regular occurrence

4. Provide feedback on direct observation only? Feedback on behaviors based on direct observation by the teacher has been reported to be more acceptable and instructive to trainees than feedback based on second-hand reporting.

5. Should the session begin with the learner’s self-assessment? A key goal of clinical training is to promote a reflective practitioner. Opening the feedback session by inviting the learner to self-assess can help achieve this goal. Use open-ended questions to start the meeting as a conversation and promote the learner’s reflection on his/her practices.

6. Reinforce positive behaviors?

7. Correct negative behaviors?

8. Use specific and neutral language to focus on performance?

9. Confirm the learner understands and facilitates acceptance? Concluded with an action plan?

10. Encouraged reflection on the skills of the faculty? Reflection by the teacher should follow every feedback session After the session ends, the teacher should reflect on what went well, what to change the next time and what new strategies he/she will adapt for future sessions.

Appendix B

MRC-01-18-136

Enrollment ID:

Gender:

PGY:

Rating Scale

**Table 4:** Rating scale

Questions	Yes	No
1. Feedback provided on direct observation?		
2. The session began with the learner’s self-assessment?		
3. Reinforced positive behaviors?		
4. Corrected the negative behaviors?		
5. Used specific and neutral language to focus on performance?		
6. Confirmed the learner understands and facilitates acceptance?		
7. Concluded with an action plan?		
8. Encouraged reflection on the skills of the faculty?		
